# Surface processes darkening the southwestern ice sheet of Kalaallit Nunaat (Greenland)

**DOI:** 10.1126/sciadv.ady9482

**Published:** 2026-07-15

**Authors:** Lou-Anne Chevrollier, Adrien Wehrlé, Joseph M. Cook, Roberts Blukis, Ian T. Stevens, Liane G. Benning, Alexandre M. Anesio, Martyn Tranter

**Affiliations:** ^1^ Department of Environmental Sciences, University of Aarhus, Roskilde 4000, Denmark.; ^2^ Department of Geography, University of Zurich, Zurich 8057, Switzerland.; ^3^ National Centre for Climate Research, Danish Meteorological Institute, Copenhagen 2100, Denmark.; ^4^ GFZ Helmholtz Centre for Geosciences, Potsdam 14473, Germany.; ^5^ Institut für Kristallzüchtung, Berlin 12489, Germany.; ^6^ Glaciology and Climate, GEUS, Copenhagen 1350, Denmark.; ^7^ Department of Earth Sciences, Freie Universität Berlin, Berlin 12249, Germany.

## Abstract

Each melt season, a “Dark Zone” develops on the southwestern margin of the ice sheet of Kalaallit Nunaat, reducing surface albedo and enhancing surface melt rates. While several processes are known to locally influence albedo, their respective contributions to the regional-scale darkening remain unknown. Here, we combine laboratory measurements, in situ observations, and physical and probabilistic modeling to quantify the albedo-reducing mechanisms across the Dark Zone. Our results indicate that the darkening is primarily driven by a combination of cryoconite material accumulation and densification of the ice column, followed by the development of microalgal blooms, while mineral dust particles do not directly contribute to the darkening. Our study provides an empirically constrained physical albedo model to represent bare ice darkening processes at play in the Dark Zone and beyond, which can guide modeling efforts to include surface darkening in ice sheet mass loss predictions.

## Teaser

Bayesian inverse modeling uncovers multifactor bare ice darkening in Southwest Kalaallit Nunaat (2017 to 2025).

## INTRODUCTION

A dark band covering an area of up to ∼10,000 km^2^, commonly called the “Dark Zone,” grows on the southwestern margin of the ice sheet of Kalaallit Nunaat (Greenland) during the summer melt season (*1*–*6*). The extent and intensity of the Dark Zone vary substantially between years (*2*, *3*), and both have followed a positive trend in the past two decades (*7*). Because of its low albedo compared to other areas of the bare-ice ablation zone, the region sustains enhanced surface melt that is not accounted for in climate models, leading to underestimations of the ice sheet runoff (*8*, *9*). Constraining the processes reducing the albedo in the Dark Zone is therefore crucial to improve models and predict the future role of the region in the ice sheet mass loss.

The low albedo of the Dark Zone was first hypothesized to be the result of meltwater accumulation at the ice surface (*1*, *10*, *11*). Subsequent examination of high-resolution satellite imagery led to the hypothesis that the darkening is driven by the presence of particles melting out from ancient ice layers (*4*). Field studies then revealed the existence of various processes influencing albedo in the region, including a fast-changing physical ice structure (*12*) and the presence of several types of particles dispersed on the ice surface (*13*–*16*). The main types of particles are pigmented microalgae (*13*, *14*); mineral fragments (*13*, *17*), hereafter referred to as mineral dust; and cryoconite (*18*), which is a microbially produced (*19*, *20*) material composed of mineral fragments and organic matter forming in situ (*21*) that often accumulate within water-filled holes.

The development of algal blooms has so far received the most attention, and the algal darkening effect was shown to be important at specific sites within the region (*14*, *15*, *22*). The high absorbency of cryoconite has been underlined in field and laboratory studies (*18*, *19*, *21*), but only the darkening effect of large granules submerged in deep holes or supraglacial streams has been quantified in the Dark Zone to date (*15*, *16*), with little consideration given to dispersed material, a much more effective albedo-reducing agent (*21*, *23*). The mineralogy of the dust has so far been found to be dominated by weakly absorbing minerals (*17*, *18*), but the darkening effect of mineral dust has not been directly quantified. Last, the densification and water saturation of the porous near-surface ice column, referred to as the weathering crust, were found to be a darkening process at least as important as the development of microalgal blooms at a field site in the south of the Dark Zone (*12*), reviving the early hypothesis that linked darkening to meltwater accumulation.

Our understanding of the albedo-reducing mechanisms in the Dark Zone has therefore improved in the past decades, but their respective roles in the large-scale darkening remain mostly unconstrained (*24*, *25*), as existing studies have primarily focused on single factors and/or specific sites. Bridging this gap requires methods that can distinguish between the different types of particles and the physical state of the ice column in the convolved optical signal acquired by satellite sensors. Physical inverse methods have proven effective at unmixing spectral signals to infer surface parameters (*26*, *27*), but the lack of physical albedo models able to describe the spectral signature of each darkening factor has so far prevented successful application of this approach.

Here, we develop a multilayer bare ice physical albedo model that incorporates empirical optical properties of the Dark Zone particles and then integrate the model into a Bayesian inversion scheme to physically quantify the effect of the different particles as well as the physical state of the ice column from spectral reflectance. We lastly apply the method to satellite imagery to evaluate the role of the different factors in driving albedo reduction in the Dark Zone.

## RESULTS

### Absorption of the Dark Zone particles

**Fig. 1.**
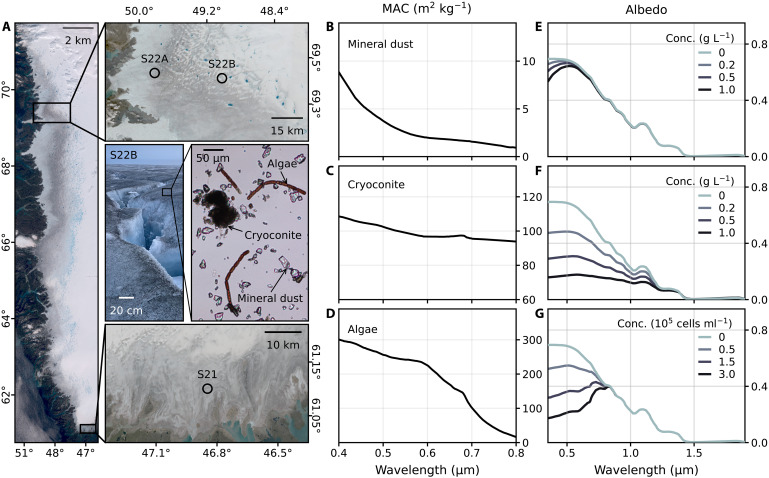
Field sites visited, absorption properties, and spectral signature of the particles found on the ice surface. (**A**) Satellite view of the southwestern ablation area (Sentinel-3 top-of-atmosphere reflectance, 22 August 2021) and the different field sites [Sentinel-2 highlights optimized RGB (red, green, and blue); S21: 23 July 2021; S22A/B: 6 August 2022], as well as ground and microscopy images from the Dark Zone site (S22B: 16 August 2022). (**B** to **D**) Mass absorption coefficient (MAC) of mineral dust, cryoconite, and microalgae (*35*). (**E** to **G**) Signature of each type of particle on the spectral albedo for increasing concentrations. L, liter.

We visited three different sites in the southwestern marginal area of the ice sheet, including the Dark Zone (Fig. 1A and fig. S1), during the melt seasons of 2021 and 2022. At each site, the ice surface primarily contained a mixture of ice microalgae, mineral dust, and dark cryoconite (Fig. 1A and fig. S1), of which absorption properties were determined and incorporated into a physical albedo model.

We analyzed mineral dust using samples from various surface types and each of the field sites (*n* = 10), because dust properties can greatly vary spatially (*28*, *29*). We found that the mineralogy was consistent between samples, with a dominance of plagioclase feldspars and quartz (table S1), corroborating previous analyses in the region (*13*, *17*, *18*, *30*). The mass absorption coefficient was also similar across samples, enabling the incorporation of a single representative mineral dust mixture into the albedo model (Fig. 1B and fig. S2). In comparison to mineral dust mixtures measured or modeled from other cryospheric environments (*29*, *31*, *32*), our samples were 2× to 5× less absorbing, supporting the prevailing consensus that the mineral dust from the southwestern margin of the ice sheet is weakly absorbing (*13*–*15*). We then determined the absorption properties of cryoconite material from in situ spectral measurements collected at S21 (Fig. 1C). The near-flat signature with a chlorophyll a feature aligns with our and previously reported observations of consistently dark opaque granules containing cyanobacteria species in the region (*13*, *18*, *33*), as well as the known dominant role of humic substances in cryoconite absorption (*18*, *21*, *34*). For microalgae, we used the in vivo absorption previously measured at S21 (Fig. 1D), which was shown to be representative of different sites in the southwestern ablation area of the ice sheet (*35*). Our data confirm the presence of a mixture of differently absorbing particles in the Dark Zone and demonstrate that each particle type produces different spectral features in the surface albedo (Fig. 1, E to G), which can be used to identify them in spectral observations.

### Inference of darkening factors from in situ spectroscopy

We developed a Bayesian inversion scheme to retrieve ice surface properties from spectral observations using the albedo model. Guided by our own—and other—field observations (*36*, *37*), we modeled the ice column as three layers with the different particles at the near-surface. The first layer represents the surface roughness, often referred to as the surface scattering layer [SSL; (*38*)], the second layer represents the remaining thicker portion of the weathering crust, and the third layer represents the unweathered impermeable ice beneath.

**Fig. 2.**
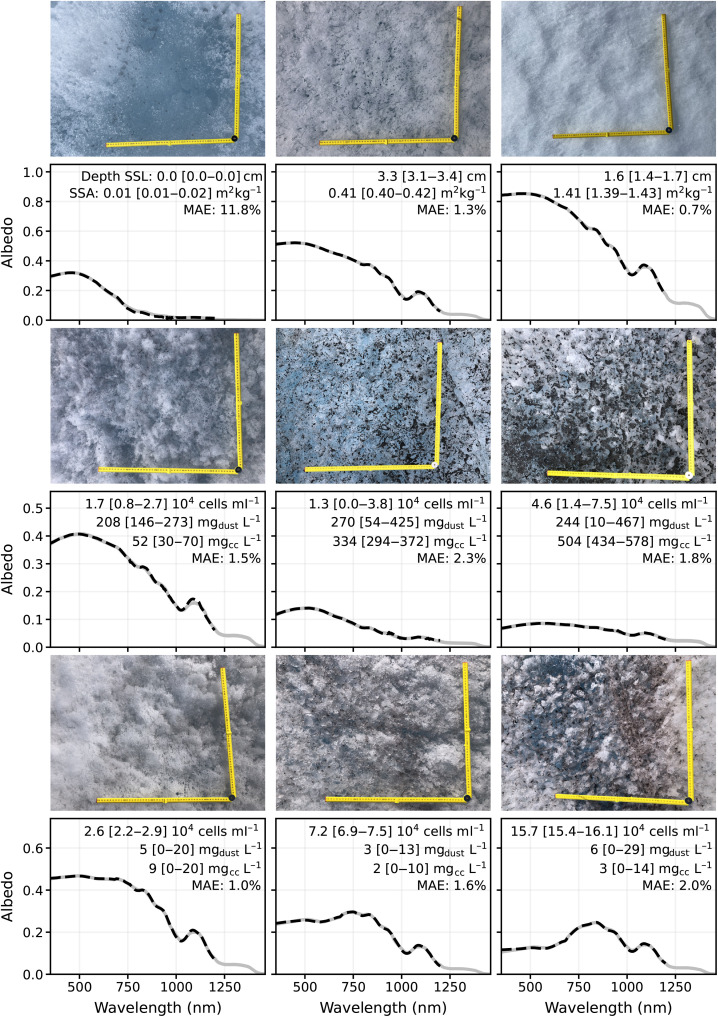
Comparison of spectral observations (black dashed curves) and model retrievals (gray curves; *n* = 4000) for various surface types. Upper panels correspond to surfaces with varying physical states and middle and lower panels correspond to surfaces with varying amounts of particles. Surface parameters are given as medians with 95% credible intervals. SSA, near-surface SSA; MAE, mean absolute error.

We applied our inversion to high-resolution spectral measurements collected at S21 and S22A and found that the model reproduced the observations with high precision (1.8% average error; max. 11.8%; *n* = 104) for a variety of spectral shapes (Fig. 2 and figs. S3 and S4) and surface conditions (tables S2 to S4), indicating that the albedo model accounts for the main mechanisms influencing the spectral reflectance of the ice surface. The largest errors (>4%) were associated with fully saturated surfaces, because the spectral features resembled more those of liquid water than solid ice and that the signal-to-noise ratio of these observations was higher than for unsaturated surfaces. We also inverted measurements collected during other field campaigns in the region (*14*, *15*) and obtained similarly low errors (1.6% average; max. 3.7%; *n* = 70).

We subsequently evaluated the inferred parameters against qualitative descriptions of the surface. The model retrieved an increasing specific surface area (SSA) as the ice surface transitioned from a saturated to fully weathered state and generally captured the presence or absence of the SSL (Fig. 2, upper panels, and fig. S4). When the ice surface looked clean, the inversion also always retrieved negligible to low concentrations of light-absorbing particles (tables S2 to S4). The model captured well the signature of cryoconite from low to high concentrations (Fig. 2, middle panels, and fig. S4), and for most spectra, the amount of cryoconite retrieved increased when the surface appeared darker. However, in <10% of the cases (*n* = 10), the retrieved concentrations deviated noticeably from expectations. Surface images indicated that half of these measurements were likely affected by shadowing, which consistently led to overestimated concentrations. For the remaining cases, deviations may be caused by situations in which the weathering crust state is spectrally too similar to cryoconite absorption, while the presence of subsurface cryoconite—ubiquitous but undetectable in surface images—may also explain higher-than-expected concentrations. In comparison, the concentrations of microalgae consistently increased as the surface appeared browner (Fig. 2, lower panels, and fig. S4), with values falling in the typically observed range (tables S2 to S4). The signature of mineral dust was mostly absent in the measurements but well reproduced by the model when present, often on surfaces with a yellow-golden tint, and for a maximum albedo-reducing effect of 0.016 [0.015 to 0.018]_95%_ (fig. S3, lower mid-panel). In contrast, the albedo-reducing effect of microalgae and cryoconite reached up to 0.12 [0.12 to 0.12]_95%_ and 0.18 [0.14 to 0.24]_95%_, respectively, and did not scale directly with particle abundance (fig. S5), as it also depends on the ice structure and the presence of multiple particle types (*27*, *35*, *39*).

In summary, the albedo model reproduced natural signatures of the ice surface with a low error, and the inversion scheme retrieved surface properties consistent with qualitative assessments, including the distinction between the different types of light-absorbing particles. However, the presence of cryoconite was more difficult to systematically distinguish from a dense ice column, as the absorption features of cryoconite are less specific than those of microalgae and mineral dust (Fig. 1, C and F).

### Regional distribution and darkening contribution of the different factors

**Fig. 3.**
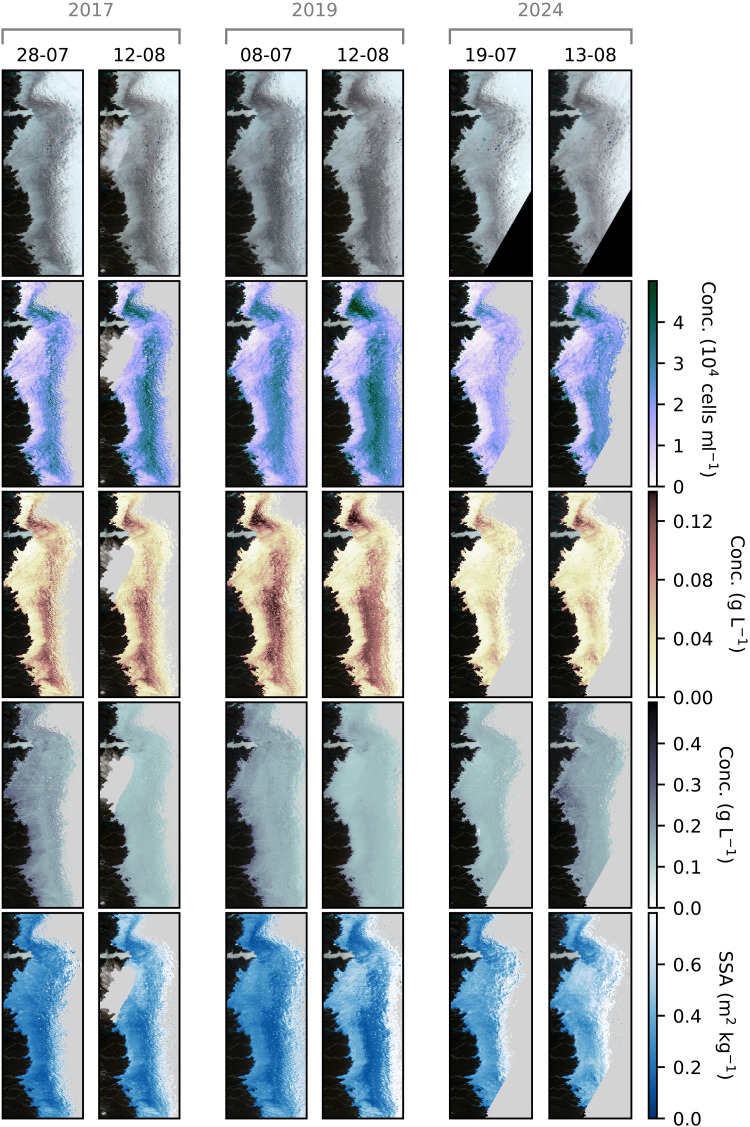
Median surface properties retrieved from Sentinel-2 imagery in July and August of 2017, 2019, and 2024. From top to bottom: algal concentration, cryoconite concentration, mineral dust concentration, and weathering crust SSA.

We applied the inversion to the available cloud-free Sentinel-2 satellite imagery covering most of the Dark Zone for the period of 2017 to 2025 (Fig. 3 and fig. S6). We observed a widespread presence of microalgae and cryoconite, with especially high concentrations in the northern region close to our S22B site, where both types of particles were very abundant in field samples (fig. S1) and microalgae were reported in high numbers previously (*22*). The range of concentrations for mineral dust was similar to our retrievals from field observations, meaning that the dust signature was weakly detectable in the satellite reflectance, likely due to its low intrinsic absorption (Fig. 1C). Cryoconite, and to a lesser extent, microalgal abundance, also coincided with the patterns of glacier foliation (fig. S7), probably because the granules consolidate around outcropping dust particles (*4*, *18*), which may also serve to fertilize algal blooms (*17*).

Algal abundance appears to increase in intensity and coverage throughout the season, especially in the northernmost third of the region (Fig. 3 and fig. S6), in alignment with previous remotely sensed estimates (*22*). The persistent presence of microalgae near the snowline also implies that the blooms are currently able to expand inland during warmer seasons and may therefore do so as the snowline retreats, especially as the cells can tolerate a wide range of surface conditions (*40*) and are not necessarily limited by nutrients (*41*).

Cryoconite abundance peaked at lower latitudes than microalgae, with spatiotemporal dynamics closely following those of the weathering crust state (Fig. 3). This latter pattern is likely driven by a combination of factors, including the coevolution of weathering crust and cryoconite holes (*42*–*44*), the accumulation of surface water caused by cryoconite-generated melt, and the difficulty to fully separate their spectral features. In comparison to microalgal blooms, the concentration of cryoconite did not exhibit a consistent increase throughout the season (fig. S6). This aligns with our understanding of cryoconite surface accumulation as being dependent on both biological processes (*19*–*21*) and fast-changing physical redistribution processes, in particular the formation and collapse of cryoconite holes (*12*, *42*–*44*).

**Fig. 4.**
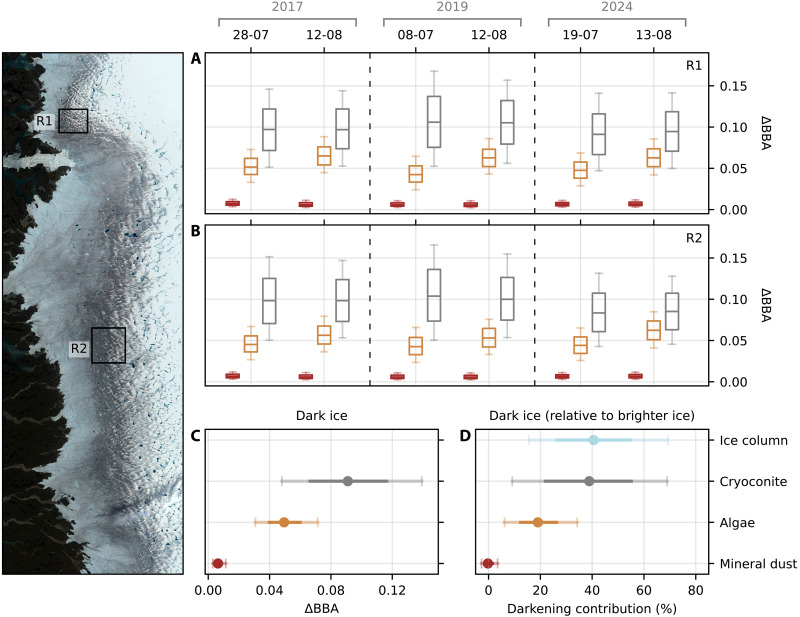
Albedo-reducing effects of the different particles and the ice column in the Dark Zone. Impact of each particle through time for two subregions on dark ice(**A** and **B**) and for all dark pixels and all dates [2017 to 2025; (**C**)]. Impact of each particle and the ice column relative to the adjacent whiter ice for all dark pixels and all dates [2017 to 2025; (**D**)]. The plots represent the median (central line/dot), the interquartile range (box/bold line), and 10th to 90th percentile range (whiskers) of the spatially averaged posterior distributions (*n* = 2000).

We calculated the albedo reduction associated with each type of particle in two subregions where dark ice was present for all dates (Fig. 4, A and B, and fig. S8, A and B), as well as for all dark ice pixels across all years (Fig. 4C). Microalgae and cryoconite consistently contributed to reducing albedo, with similar temporal variabilities as in their respective abundances, whereas mineral dust had a negligible influence across space and time. The effect of microalgae relative to cryoconite also varied in time but remained smaller, averaging ∼2× lower at the scale of the Dark Zone (Fig. 4C).

To evaluate how the different particles as well as the ice structure contribute to the regional-scale darkening, we ultimately computed their albedo-reducing effects relative to the adjacent brighter ice conditions across all years (Fig. 4D). Our results indicate that a combination of cryoconite accumulation and ice column densification drives the majority of darkening (81% [65 to 93%]_80%_), which are both factors that have been undermined so far in the narrative of the Dark Zone. The growth of microalgal blooms explains the remainder of the darkening (19% [5 to 33%]_80%_) and is therefore a notable contributor at the regional scale and, in particular, in the northern region.

## DISCUSSION

Our study provides an empirically constrained physical albedo model to simulate bare ice darkening processes occurring in the Dark Zone and beyond, as well as a Bayesian inversion scheme to disentangle their respective effects. We used this framework to provide constrains on the contribution of each factor in the Dark Zone, thereby unifying existing hypotheses into a coherent and data-driven understanding of the region’s surface darkening.

Our calculations quantify the direct radiative impact of light-absorbing particles but do not account for their indirect effects. On bare ice, light-absorbing particles contribute to the densification or saturation of the ice structure via the meltwater they produce, in the same way that dust triggers metamorphism in snow (*45*). They can also prevent the (re)formation of the weathering crust by absorbing a substantial part of the incoming short-wave radiation that would otherwise cause internal melting and weathering (*37*). These effects are attributed to the ice column in our study, and so, our calculations of cryoconite and microalgae contributions to the overall darkening are therefore likely conservative.

Substantial uncertainties remain in our estimates, with the dominant sources of error pointing to practical directions for future work. An important part of the uncertainties originates from the satellite data itself, in particular the atmospheric correction required to derive surface reflectance and the low spectral resolution of Sentinel-2 imagery, which limits the ability of the algorithm to identify specific spectral features. The acquisition of hyperspectral airborne data may provide additional constraints—albeit over restricted spatial coverage—whereas an alternative approach would be to incorporate the atmosphere directly into the inversion scheme and optimize top-of-atmosphere satellite reflectance (*46*). This would also enable the use of satellite imagery with higher temporal resolution than Sentinel-2, such as Sentinel-3 imagery, to better constrain the seasonal and interannual variability in the darkening factors and the associated meteorological/phenological drivers.

Our analyses demonstrate that the spectral similarity between cryoconite particles and a dense weathering crust is another important source of uncertainty and more generally presents a challenge for spectroscopic analyses. In the context of the method we present here, this uncertainty may be reduced by imposing tighter constraints on the prior knowledge for the inversion, as well as reducing the range or number of parameters in the albedo model configuration. This would require new in situ data directly relating measurements of surface parameters to spectral observations, ideally in the central region of the Dark Zone, from where no published documentation of the state of the surface exists. These new data may also uncover marginal additional darkening processes that are not currently accounted for in the model, such as the presence of snow algal cells retained from the melted snowpack, which we and others (*47*) occasionally observed on bare ice (fig. S1).

Looking into the future, the multifactor nature of the ice surface darkening means that various biological, chemical, and physical mechanisms—as well as their interactions—must be considered to predict the development of the Dark Zone. Ultimately, mechanistic models representing weathering crust evolution, cell growth, transport, and retention (*48*) as well as models describing cryoconite hole development (*43*) could be calibrated in the Dark Zone and coupled to our albedo model, enabling a physically based implementation of the Dark Zone in regional climate models.

## MATERIALS AND METHODS

### Field sampling and measurements

#### Surface particulate collection

Three different locations within the southwestern ablation area of the ice sheet of Kalaallit Nunaat (Greenland) were visited during the summer melt seasons of 2021 and 2022 [19 July to 9 August 2021 (S21): 61.100428°N, −46.847007°E; 17 July to 16 August 2022 (S22A): 69.43137659°N, −49.86703194°E; 16 August 2022 (S22B): 69.41145°N, −49.021196°E; Fig. 1A]. Surface particulates were collected in sterile Whirl-Pak bags from three different surface types: snow, ice, and cryoconite holes. The samples were left to melt at ambient temperature after collection, then poured into several Falcon tubes, and frozen at −20°C until laboratory analyses.

#### Ground spectroscopy

Hemispherical-conical reflectance factor measurements were acquired at both the S21 and S22A locations (Fig. 1A) using an ASD Fieldspec4 (spectral range: 350 to 2500 nm) and a black tripod, following standard methods (*49*). We selected surfaces that were as homogeneous as possible, then a ruler was used to align the fore-optic to the middle of the target surface, and surface pictures were taken with the ruler so that the center of the ruler corresponds to the center of the measurement footprint (9- to 16-cm diameter). Each measurement was acquired with a collimated lens of field of view of 10° in 10 replicates, immediately (<10 s) after measuring a reference spectrum using a calibrated Spectralon panel. All measurements were performed at nadir view (viewing zenith angle θ = 0°). We removed the spectra with obvious calibration errors as well as the ones exhibiting a null signal in the infrared and/or a very high signal-to-noise ratio. In total, 104 spectra were used from S22A and S21, and 70 additional spectra previously collected around station S6 in the K-transect in 2014 (*14*) and 2016 to 2018 (*15*) were added to the analysis. All spectra were corrected for the step at 1000 nm caused by the misalignment of the short-wave infrared and near-infrared sensors in the ASD FieldSpec (*50*).

### Laboratory analyses

#### Sample processing

The samples were treated in the laboratory to isolate the inorganic (mineral) material by repeated hydrogen peroxide reaction at 60° to 70°C until effervescence stopped and the biological material was oxidized (*17*, *21*). After treatment, the samples were rinsed, freeze dried, and split in two for mineralogy and spectrophotometry analyses.

#### Spectrophotometry

The freeze-dried samples were resuspended, and the particulate absorption coefficient (Ap_λ_, m^−1^) was determined with the filter-pad transmission method (*51*) using a spectrophotometer equipped with an integrating sphere (Shimadzu 2700, 2600-ISR). Details of the spectrophotometric measurements are provided in Supplementary Text. The inorganic dry mass concentration (kg m^−3^) of the analyzed samples was determined by filtering a known volume onto three preweighed GF/F filters, combusting the filters, reweighing them, and normalizing to the volume. Ap_λ_ was then normalized to the inorganic dry mass concentration to yield a mineral dust mass absorption coefficient (m^2^ kg^−1^).

#### X-ray diffraction

X-ray diffraction measurements were performed with a STOE STADI P diffractometer with a Cu x-ray source fitted with a curved Ge(111) monochromator (Kα_1_ = 1.5406 Å) and a DECTRIS MYTHEN2 R detector in a flat plate transmission geometry. Diffraction patterns were measured over a range of 0° to 96° 2θ (*Q* = 0 to 6.06 Å^−1^) for a duration of 0.5 hours per data point. Patterns were analyzed by Rietveld refinements to quantify the mineralogy, which were performed using GSAS-II (*52*). Reference CIF files were obtained from the AMCSD database. During the refinement unit cell, the grain size and microstrain were optimized. For major minerals (minimum wt % >20%), thermal displacements and chemical composition were also optimized if relevant. The background was approximated using Chebyshev polynomials, and the instrument function was calibrated by empirically fitting the Caglioti function with included asymmetry to a National Institute of Standards and Technology Si measured in the same geometry.

### Remote sensing

Sentinel-2 and Sentinel-3 imagery was downloaded from Sentinel hub services using the earthspy Python package (*53*). Sentinel-3 data were solely used to illustrate the location of the study area, while the Sentinel-2 L2A product was used for analysis. The band 9 of Sentinel-2 L2A at 945 nm was empirically corrected for the bias resulting from water vapor by building a correlation between band 8A and band 9 calculated from the field spectra (*n* = 174, *r*^2^ = 0.99; slope: 0.7868; intercept: −0.0057). Dark ice was defined using a reflectance threshold of 0.45 on the band 4 of Sentinel-2 data (*3*). Supraglacial lakes were masked using normalized difference water index thresholds of 0.25 on dark ice (*54*) and 0.35 on brighter ice, and snow was masked using a reflectance threshold of 0.7 on band 4. Both thresholds were chosen from visual inspection of the imagery so as to remove any pixel within lakes as well as flooded snow. Clouds and margins were masked manually.

### Radiative transfer modeling

#### Model development

We developed SNICAR-fx (https://github.com/openosmia/snicar-fx), a Python-based open-source radiative transfer model for snow and ice albedo, on the basis of and extending SNICAR-ADv4 (*55*, *56*) and BioSNICAR (*15*). The model solves the radiative transfer equation for unpolarized light, considering the ice column as plane parallel and made of one or several homogeneous layers defined by their inherent optical properties, which are calculated by combining the single scattering properties of the snow/ice medium and light-absorbing particles. SNICAR-fx computes the snow/ice single scattering properties with geometric optics rather than Mie theory and offers additional features such as a flexible spectral range and resolution, a multistream solver, a lightweight format, a fast implementation, the presence of liquid water within ice/snow, and empirical optical properties of bare ice light-absorbing particles presented in this study. We chose a bulk approach to incorporate the light-absorbing particles into the model rather than a single particle approach because the particles are often clumped and mixed together at the ice surface, violating the assumption of scattering field independence of commonly used methods such as Mie theory. The mineral dust and algal cells were incorporated by directly altering the absorption coefficient of the ice medium with the measured particle absorption coefficient. The optical properties of cryoconite material were inferred from two independent reflectance measurements of optically thick layers of cryoconite made under diffuse conditions at S21. More specifically, the measurements were manually inverted with SNICAR-fx, assuming a constant asymmetry parameter of 0.8, and then inserted into the model.

#### Model configuration

SNICAR-fx was configured to represent the weathering crust system reported from observations during field campaigns on the ice sheet, which consists in a thin upper layer of loosely bound ice crystals with varying amounts of impurities and a progressively denser layer of weathered ice with a varying level of water saturation (*36*). We modeled this system using a three-layer ice column made of an upper highly scattering granular layer of varying thickness and SSA, a middle weathering crust layer with lower scattering and varying thickness and SSA, and a third layer of even lower scattering to reach a 10-m depth in total, below which all light is assumed to be extinct. We included liquid water in the two upper layers to capture the signature of melting surfaces and made it to vary with density (10% of the total density). We set the density of the last layer to 910 kg m^−3^, which was recently defined as a boundary between the weathering crust and the impermeable ice (*37*). The effective radius of air bubbles in each layer decreased with depth (1500, 5000, and 35,000 μm), and the values were chosen to match existing empirical relationships between the density and SSA (*57*). The different light-absorbing particles were placed in the upper 5 mm of the ice column, and a Fresnel interface was placed between the upper granular layer and the second layer to account for the change in refractive index. We did not include black carbon in the model because the maximum concentrations ever measured in the region are so low (*14*, *58*, *59*) as to not substantially contribute to albedo reduction (*14*). For the ice absorption coefficient, we used empirical measurements from the southwestern ablation area of the ice sheet (*60*), and for the liquid water absorption coefficient, we used recent measurements at 0°C (*61*). We set solar zenith angle (SZA) at 50°, which represented an average value for the time and location at which the ground spectroscopy and satellite data used in this study were acquired. The SZA of the satellite images varied between 46° and 54°, which produces minor variations in the surface albedo (σ = 0.009 on average in the inversion spectral range) in comparison to the effect of surface properties. The model spectral resolution was set to 5 nm in the range of 300 to 2500 nm, where solar irradiance is concentrated and most spectroradiometers operate. We used the two-stream solver of SNICAR-fx to solve the radiative transfer equation and simulate spectral albedo, which is based on the Delta-Eddington solution to solve for the multiple scattering within and between each layer, and an adding-doubling solver to combine the layers (*55*, *56*). We lastly calculated albedo [broadband albedo (BBA)] from the spectral albedo using the subarctic surface irradiance spectrum (SZA = 50°) computed with SWNB2 and available in SNICAR-ADv3 (*29*).

#### Deep learning emulator

A deep learning feed-forward neural network emulator of SNICAR-fx was built with seven input features (surface parameters), 440 target features (spectral bands), and 12 layers (290,383 trainable parameters and exponential linear unit activation function) on the basis of a previously developed architecture for a snow albedo model (*27*). In total, 10.5 million SNICAR-fx simulations were run for random input variables in the following ranges: algal concentration (0 to 5 × 10^5^ cells ml^−1^), cryoconite concentration (0 to 5 g liter^−1^), mineral dust concentration (0 to 10 g liter^−1^), SSL depth (0 to 15 cm) and SSA (0 to 5.8 m^2^ kg^−1^), weathering crust depth (0 to 2.5 m), and SSA (0 to 1.75 m^2^ kg^−1^). All parameters were normalized between 0 and 1 before training to ensure that equal weight is given to each parameter during training. A 90/10 split of this dataset was used for the training and testing sets. The model was trained for 400 epochs with an adaptive learning rate decreasing exponentially from 0.001 and a batch size of 256. For each epoch, the training dataset was shuffled, and 10% was used for validation. After training, the mean error on the spectral albedo on 99% of the testing test was under 1.2 × 10^−4^, with the largest errors associated with extreme input values that correspond to the boundaries of the parameter ranges and are unrealistic for natural conditions. The average errors on the entire training and testing sets were both 3 × 10^−5^, overall indicating that the emulator does not overfit and reproduces physical simulations very accurately.

### Bayesian inverse modeling

#### Inference algorithm

We developed a sampling-based Bayesian inference framework to retrieve ice surface properties and associated uncertainties from spectral observations using the SNICAR-fx emulator. We used the No-U-Turn sampler (*62*) as implemented in TensorFlow Probability version 0.25 (*63*) using dual-averaging adaptation with an initial step size of 0.1 and a target acceptance probability of 0.75. Each retrieval was run with four independent chains. For ground reflectance data, we used 1000 burn-in and 1000 posterior samples per chain, and for satellite observations, we used 500 and 500, respectively, as convergence was reached more rapidly because of the lower spectral resolution. The adaptation period was set equal to the burn-in. All parameters were constrained to their physically valid domains, i.e., positive values within the range of the emulator, and the SSL depth was additionally constrained between 0 and 7 cm, as the air-ice transition zone is typically only a few centimeters thick. We used uniform priors for the ice SSA and a Beta(2, 3.5) prior for the layer depths, which provides a broad distribution only mildly down-weighting depths approaching the upper bound, corresponding to 1.2 m for the weathering crust (*36*). We used a Beta(0.95, 3) prior for light-absorbing particle concentrations, empirically designed to distribute probability mass across several orders of magnitude. A Student-*t* likelihood with ν = 3 was chosen to enhance robustness to outliers in the measurements. Initial values were randomly drawn from the priors, except for light-absorbing particle concentrations, which were initialized randomly within the first quartile of their priors as we found that it accelerated convergence. The resulting posterior distributions provide estimates of particle concentrations as well as the SSA and depth of each layer. The darkening effects were calculated by differencing the BBA to the BBA calculated with altered surface conditions (e.g., particle concentration set to 0). We note that the inferred particle concentrations depend on the visibility to the satellite sensor, as is inherent to optical remote sensing. They therefore represent apparent concentrations that are directly related to radiative impacts, in line with our objective to quantify darkening effects.

#### Application to ground and satellite reflectance

Before inversion, field reflectance spectra were resampled from 1- to 5-nm resolution to match the emulator resolution. For satellite data, the emulator simulations were resampled to Sentinel-2 bands directly in the likelihood calculations using the instrument spectral response. We performed the inversion on clusters of satellite pixels rather than on every individual pixel, which would have been computationally prohibitive. Clusters were defined so that the maximum distance within a cluster was smaller than the observational uncertainty (see below), ensuring that the intracluster variations were not physically meaningful. Each individual pixel was then mapped to its closest centroid. This procedure both reduced computational cost and prevented overinterpretation of noise in the retrievals. We excluded marginal sloped areas in the clustering and used a total of 4900 clusters, explaining 99% of all pixels within observational uncertainty.

We adopt the perfect model assumption and explicitly account for errors in the data. For field spectra, we estimated the standard deviation across the 10 replicates and used an upper bound of 0.004. For satellite data, we derived standard deviations for dark and brighter ice (see the “Remote sensing” section) using the L2A RUT tool (*64*). We included the error related to adjacency effects and the atmospheric correction but not the Lambertian assumption in the atmospheric correction, as its effect is unknown and the study region is relatively flat (see below). We then extracted 75 clusters within the dark ice and 75 others within the brighter ice from scenes covering the beginning, middle, and end of the season and averaged their standard deviations to yield a representative error for dark ice and brighter ice separately. Our approach considers the ice surface Lambertian, an assumption that has been used in other studies with similar applications (*26*, *35*). We consider this hypothesis justified because the region of interest is relatively flat compared to mountainous areas, and the bidirectional reflectance function (BRDF) of the weathering crust is unknown. Given that the weathering crust scattering phase function remains undetermined, the BRDF cannot be accurately modeled with multistream radiative transfer models at present, and arbitrarily deciding on a function could introduce further errors. In practice, the BRDF is also likely surface-specific because weathering crust surfaces exhibit high variability in terms of structure and spectral signature, and therefore, the correction to be applied to measurements is unlikely unique. To mitigate the error associated with anisotropy, we restricted the inversion in the spectral range below 1200 nm, where snow anisotropy is known to be less important (*65*), and used spectral measurements collected at nadir view close to solar noon.

We visually inspected the trace plots, posterior distributions, and errors for all chains of each field measurement and found that all chains converged well to the exact same solution for 165 of 174 observations (95%). For the other measurements (*n* = 9), three of them corresponded to fully saturated surfaces with very low reflectance so that the spectral features of ice were too obscured for the model to properly separate them and the sampler converged to two modes. The other two had one chain converging to an unrealistic solution with high residuals, indicating a potential initialization issue, so we discarded that chain. The other two only had one chain converging to a well-constrained posterior, and we retained that chain. The final two measurements oscillated between two well-constrained posteriors and were associated with a shadowing issue, as discussed in the main text. For the satellite retrievals, it was not feasible to visually inspect each chain in detail as we did for the field measurements, so we relied on summary diagnostics computed with Arviz Python package version 0.22.0 (*66*). We calculated the rank-normalized, split-chain $\hat{R}$ (*67*), which was, on average, lower or equal to 1.006 for all parameters and lower than 1.017 for 99% of the samples. We also calculated the bulk and tail effective sample sizes, which were, on average, higher than 500 and 450, respectively, for all parameters. While the satellite retrievals had much broader posteriors than the field measurements because of the higher observational noise and lower spectral resolution, the diagnostics therefore indicate satisfactory convergence, mixing of the chains, and exploration of the posterior space (*67*). Last, we computed Bayesian credible intervals as high-density intervals to represent uncertainty, which corresponds to the shortest interval containing the specified posterior probability reported in the text.

## Supplementary Materials

### This PDF file includes:

Supplementary Text Figs. S1 to S8 Tables S1 to S4 References
