# Supplementary material for: Surface processes darkening the southwestern ice sheet of Kalaallit Nunaat (Greenland)
Source: Sci Adv. 2026 Jul 15;12(29):eady9482. doi: 10.1126/sciadv.ady9482 (PMC13371923; doi:10.1126/sciadv.ady9482)
Supplement: Supplementary file 1 — Supplementary Text Figs. S1 to S8 Tables S1 to S4 References [file sciadv.ady9482_sm.pdf]

Science Advances
AAAS

Supplementary Materials for

Surface processes darkening the southwestern ice sheet of Kalaallit Nunaat (Greenland)

Lou-Anne Chevrollier *et al.*

Corresponding author: Lou-Anne Chevrollier, lou.chevrollier@envs.au.dk

Sci. Adv. **12**, eady9482 (2026)
DOI: 10.1126/sciadv.ady9482

This PDF file includes:

Supplementary Text
Figs. S1 to S8
Tables S1 to S4
References

Supplementary Text (Methods)

Spectrophotometry

The spectrophotometer was first baselined with a clean wet GF/F filter inserted between two glass slides at the entrance of the sphere. A transmission spectrum of this filter was measured to ensure that the baseline was correct. Then, a known volume of resuspended sample was carefully filtered onto that filter, ensuring homogeneous distribution on the filter. Three to six transmission spectra of the sample were measured in a similar way as the blank filter. The filter was positioned in the same orientation than for the blank scan for two spectra, and then the filter was rotated to account for within-sample variability. The spectra showing signs of instrument drifts such as transmission above 100% were discarded and the particulate absorption (Ap_λ, m^{-1}) was calculated from the transmission spectra (*51*):

$$Ap_\lambda = \ln(10) \times 0.679 \times \log\left(\frac{T_\lambda}{100}\right)^{1.2804} \times \frac{A}{V}$$

With A the area covered by the sample on the filter (m^2), λ the wavelength (400 - 700 nm), V the volume of suspension filtered (m^3), and T_λ the transmission spectrum of the sample (%). Since this method is prone to backscattering biases (*51*), the particulate absorption is typically post-corrected with a null-baseline correction in the near-IR (750nm), assuming that the sample does not absorb beyond the selected wavelength and that the scattering is wavelength-independent. Several studies however detected absorption beyond 750nm on different types of water sediments (*68–70*), as well as mineral dust found in snow (*31*). Since hornblende was present in the mineralogy, we considered here that the null-point correction may not be valid. Instead, we corrected for backscattering by applying a half null-point correction at 750 nm to T_λ, following experimental results (*68*) showing that 50% of the transmission at 750 nm could be attributed to scattering error in inorganic sediment samples.

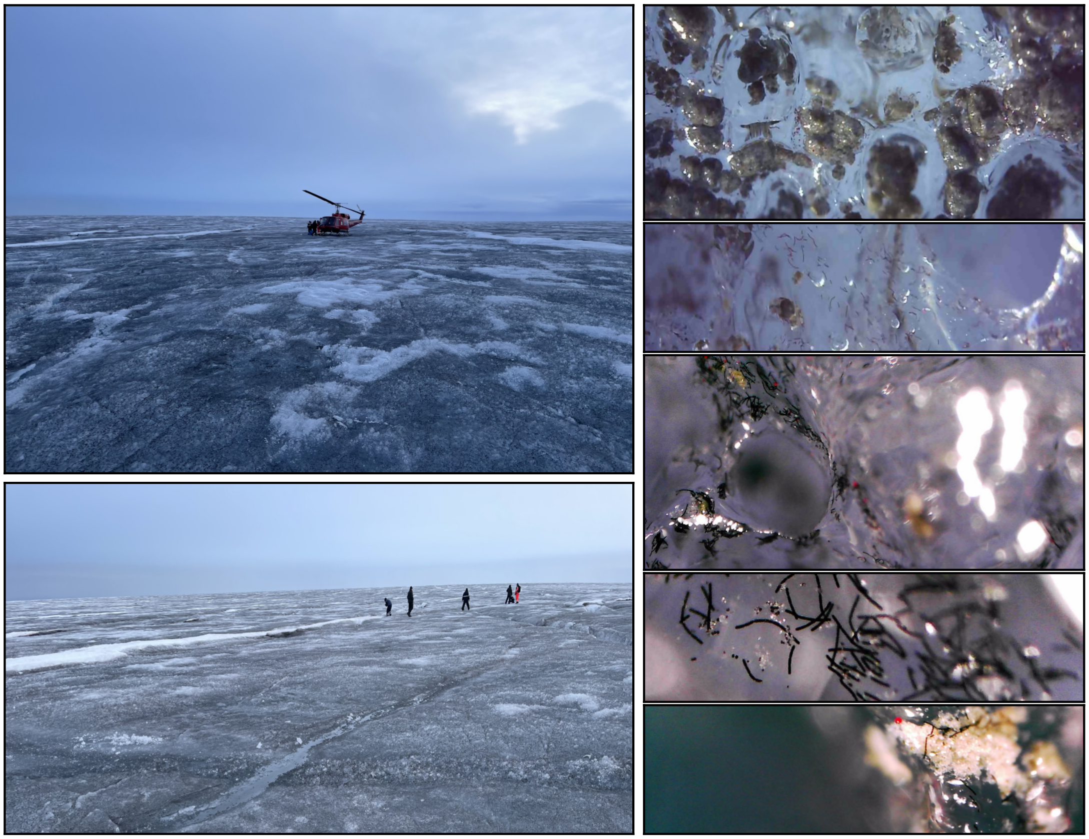

Fig. S1. Surface and hand-held microscope pictures from the site S22B on August 16th 2022.

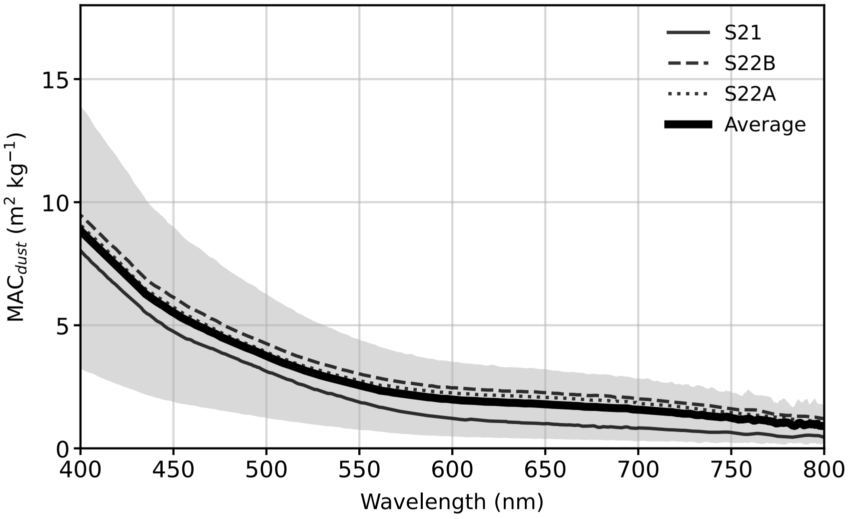

Fig. S2. Mineral dust absorption between sites.

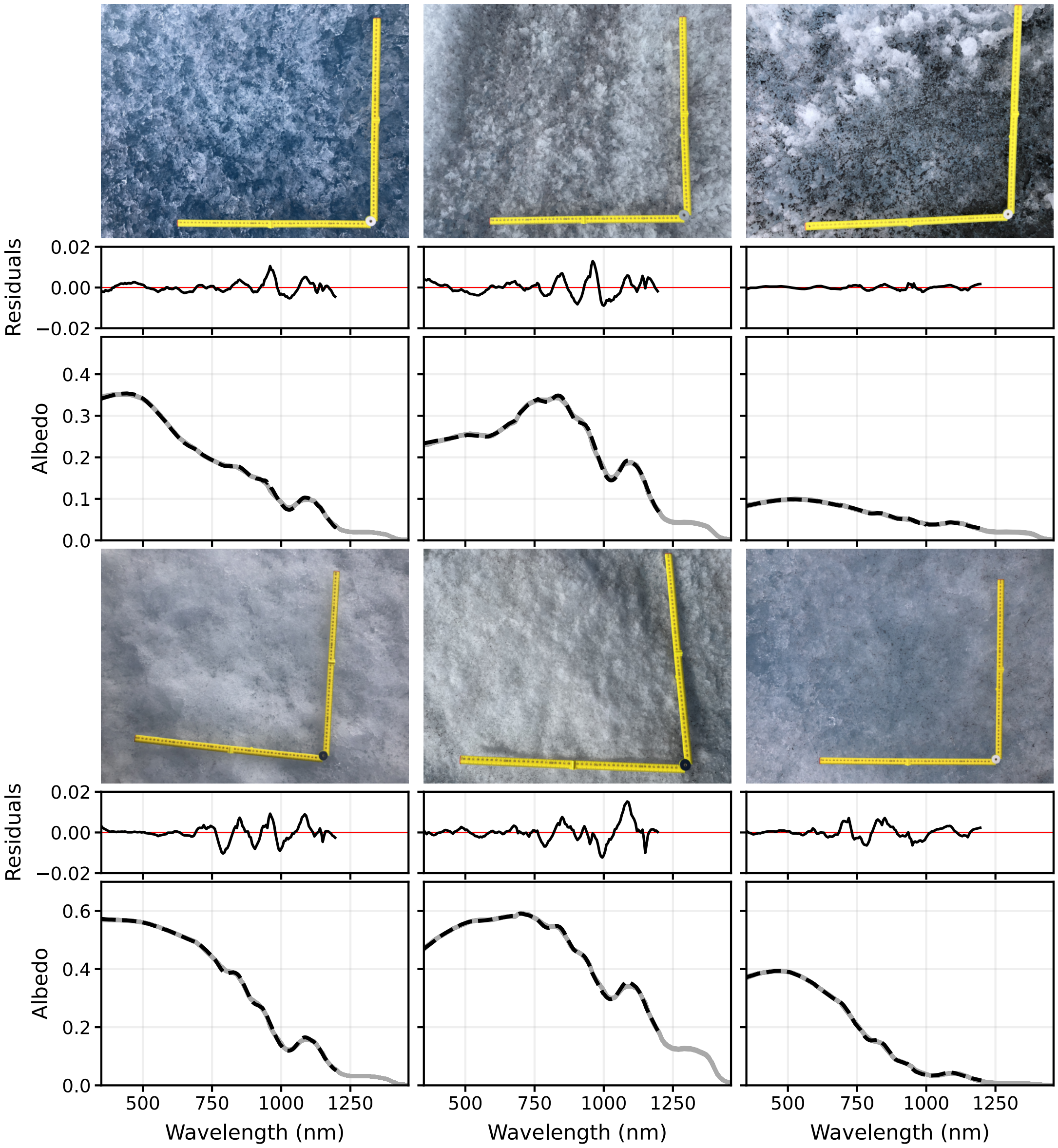

Fig. S3. Comparison of spectral observations (black curves) and model retrievals (gray curves, n=4000) for various surface types, along with the residuals corresponding to the difference between observations and model simulations.

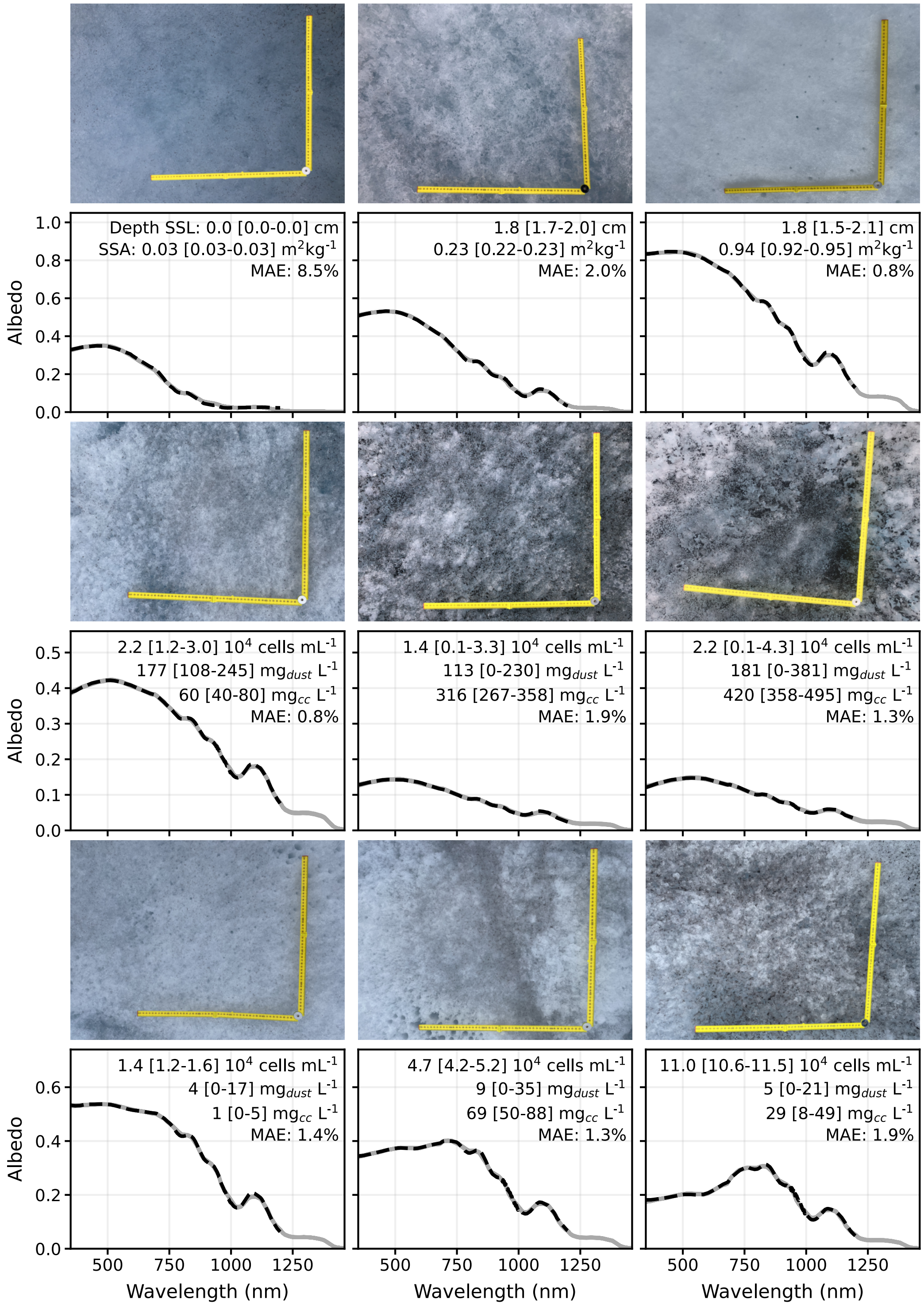

Fig. S4. Comparison of spectral observations (black dashed curves) and model retrievals (gray curves, n=4000) for surfaces with varying physical states (upper panels) and amounts of particles (middle and lower panels). Surface parameters are given as medians with 95% credible intervals. SSL: surface scattering layer, SSA: near-surface specific surface area.

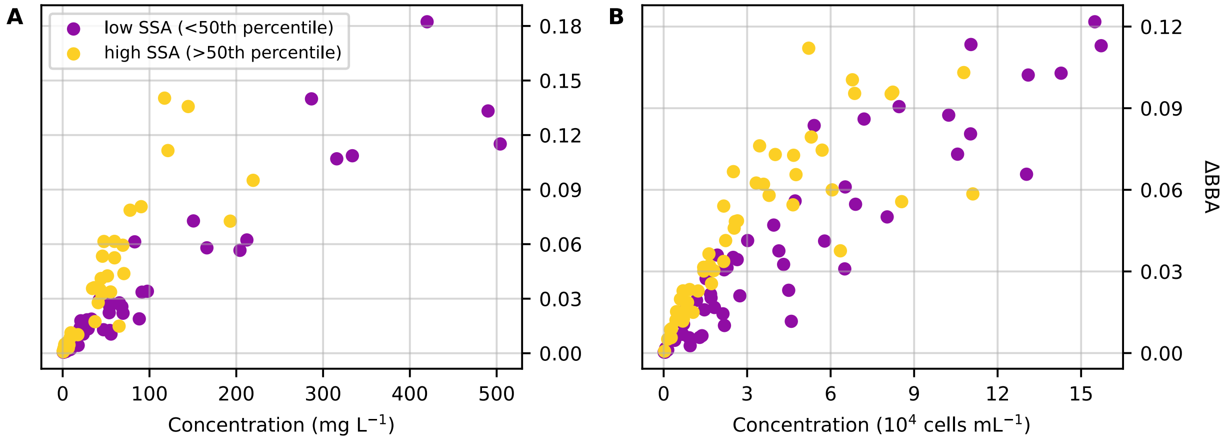

Fig. S5. Relationship between the abundance and albedo-reducing effect of cryoconite (A) and microalgae (B), illustrating the effect of the ice structure on the darkening efficiency of particles.

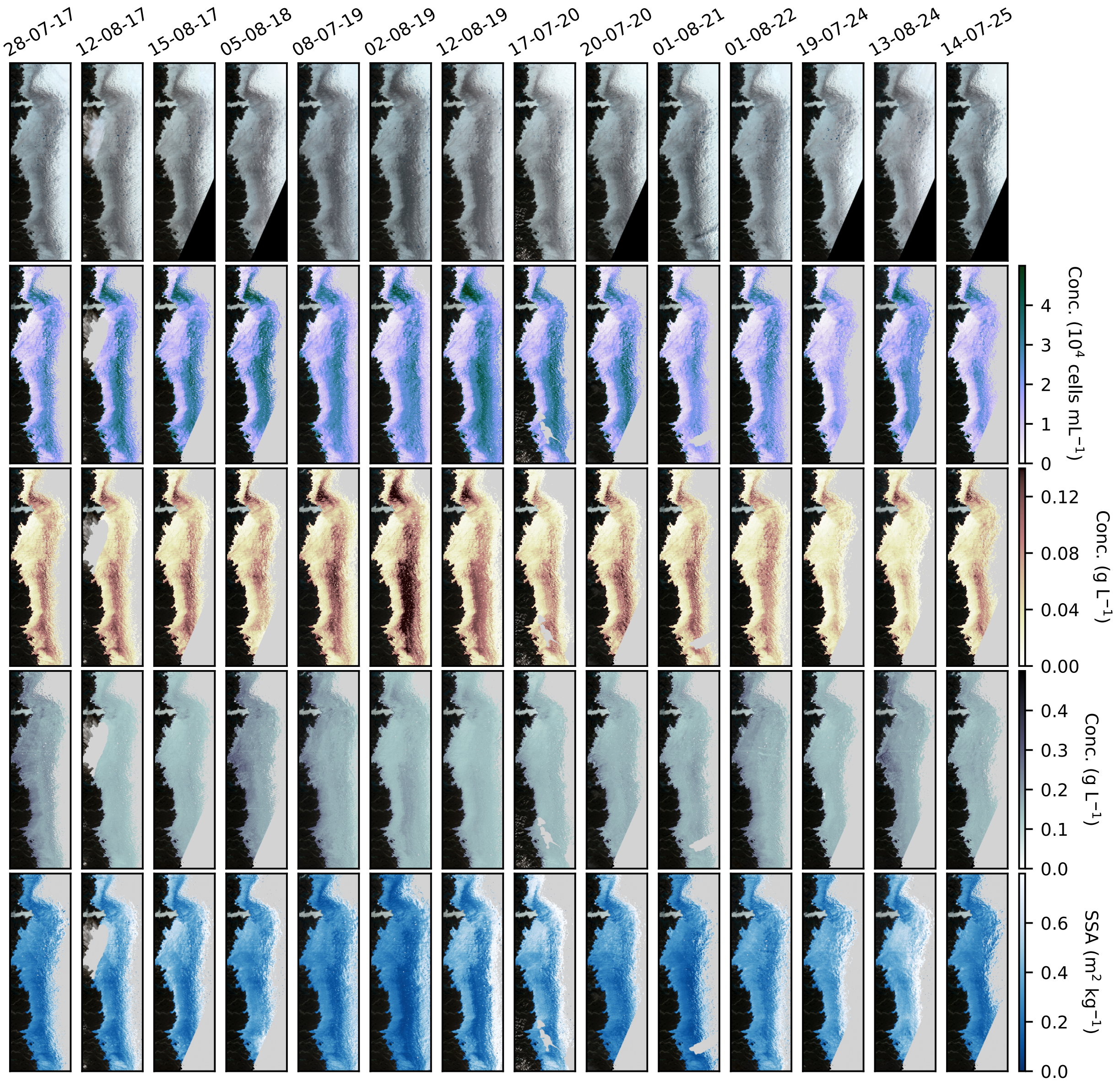

Fig. S6. Median surface properties retrieved from Sentinel-2 imagery (July and August in the period 2017-2025). From top to bottom: algae, cryoconite, mineral dust, and weathering crust specific surface area.

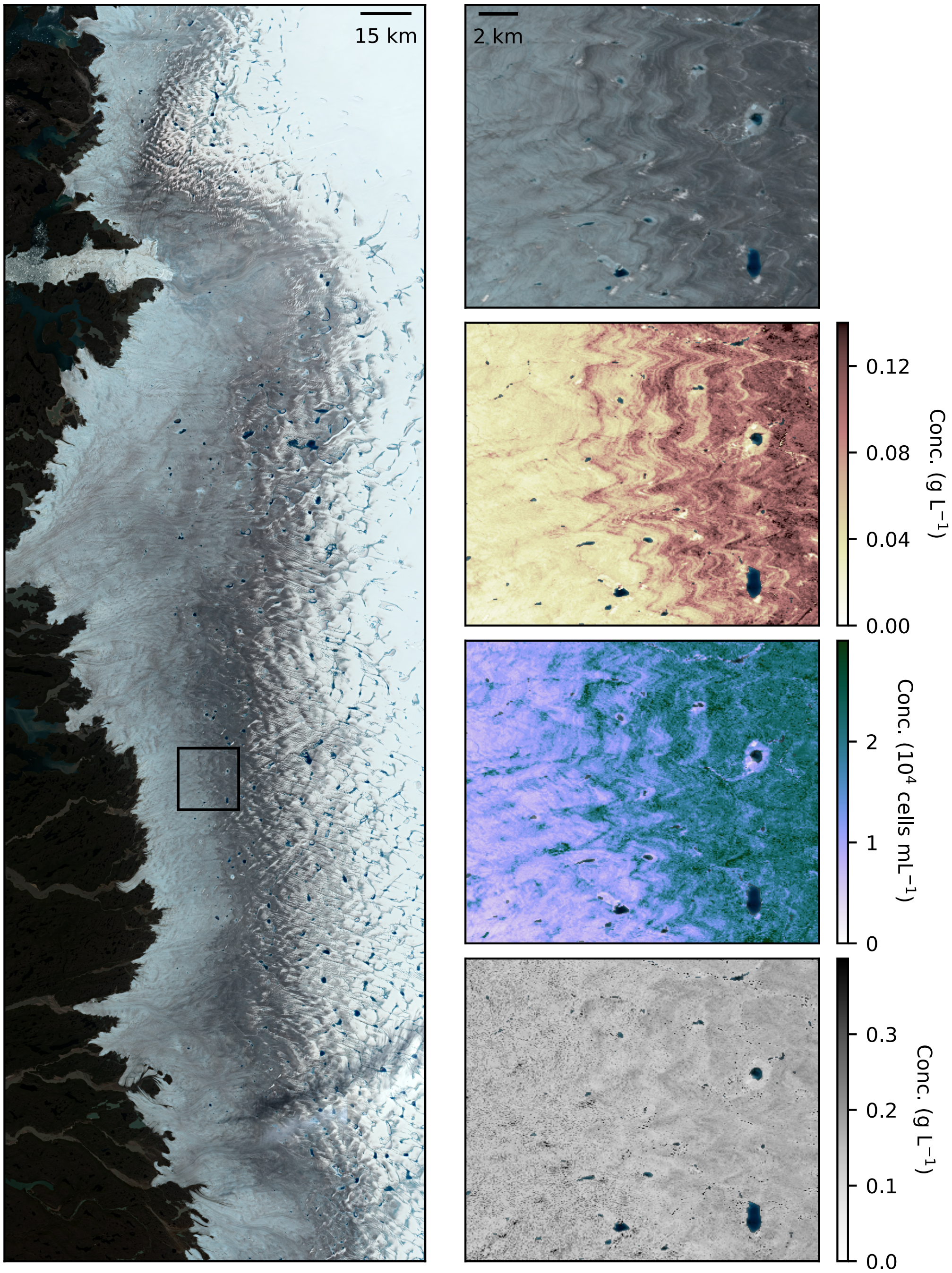

Fig. S7. Illustration of the association between particle abundance and glacier foliation patterns.

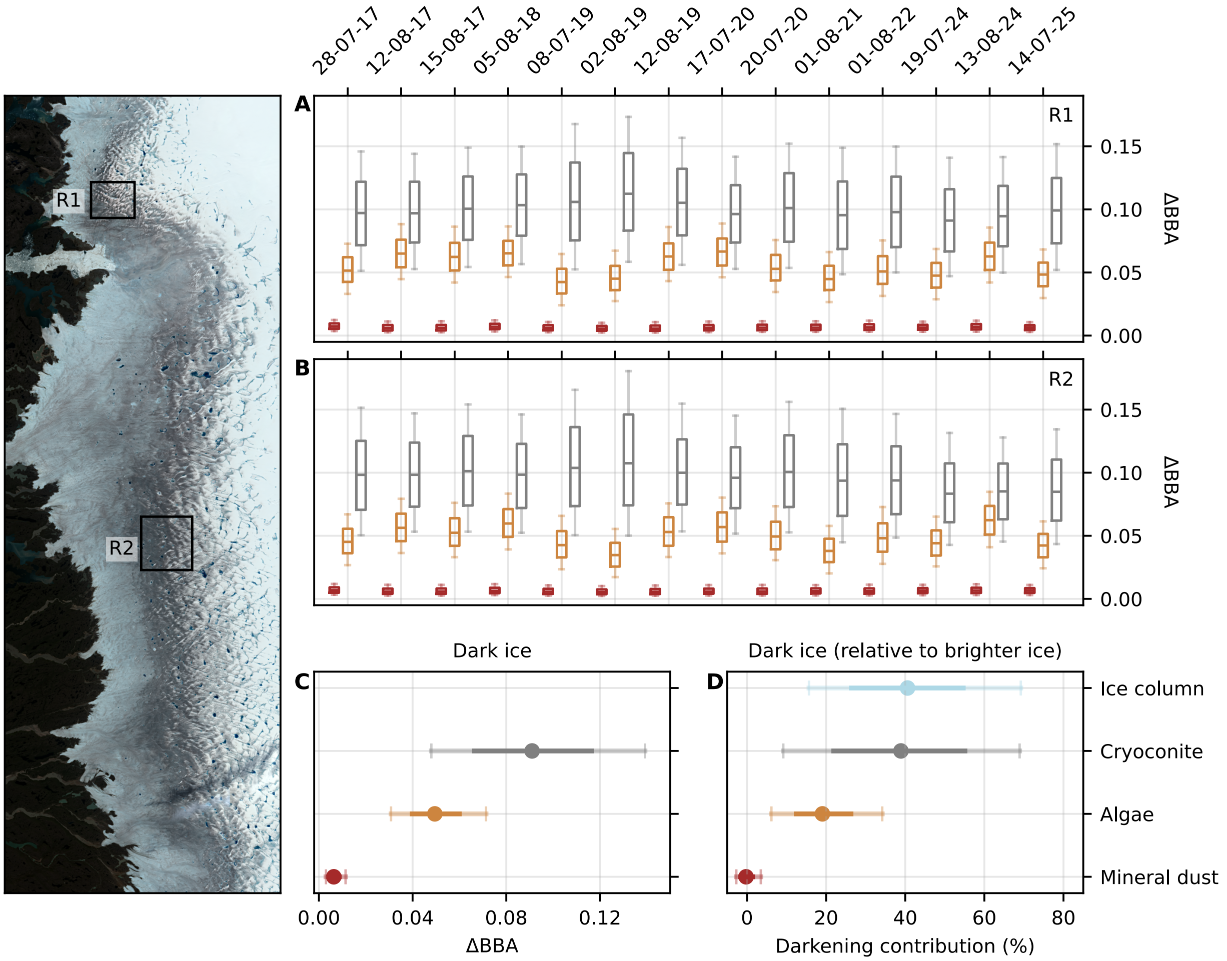

Fig. S8. Albedo-reducing effects of the different particles through time for two sub-regions (A, B), for all dark pixels and all dates (2017-2025; C), and for all dark pixels and all dates relative to the adjacent whiter ice (2017-2025; D). The plots represent the median (central line/dot), the interquartile range (box/bold line) and 10-90th percentile range (whiskers) of the spatially-averaged posterior distributions (n=2000).

Table S1. Mineral composition in weight percentage of the different samples, along with the weighted residual error factor (Rwp, %). Sample ID indicates field site (S22A, S22B, S21), habitat (CH, SN, SI: cryoconite holes, snow, surface ice) and treatment (H2O2, raw, burnt).

Sample ID	Quartz	Albite	K-feldspars	Hornblende	Augite	Entstatite	Muscovite	Chlorite	Vermiculite	Hematite	Magnetite	Apatite	Dolomite	Calcite	Total	Rwp
S22A-CH-raw	19.1	48.6	6.3	20.3	0.8	0.8	2.1	1.1	0.4	0.0	0.0	0.1	0.2	0.1	99.9	4.8
S22A-CH-H2O2	15.7	32.1	36.0	13.4	0.4	0.4	1.2	0.4	0.0	0.0	0.1	0.0	0.2	0.1	100.0	9.1
S21-CH-raw	15.3	57.4	17.9	4.7	0.2	0.0	2.6	1.5	0.3	0.0	0.1	0.0	0.2	0.0	100.2	7.1
S21-CH-H2O2	19.2	43.2	26.6	5.7	0.4	0.0	3.6	0.8	0.2	0.0	0.1	0.0	0.0	0.0	99.8	4.7
S22B-SI-H2O2	33.5	50.8	9.4	3.8	0.2	0.2	1.7	0.3	0.0	0.0	0.0	0.0	0.0	0.0	99.9	11.3
S22B-SI-raw	28.3	49.7	7.7	8.3	1.1	1.8	1.9	1.0	0.2	0.1	0.0	0.0	0.0	0.0	100.1	7.3
S22B-SI-burnt	30.9	51.7	10.9	4.3	0.4	0.1	1.3	0.2	0.0	0.0	0.0	0.0	0.0	0.1	99.9	7.9
S21-SI-a-raw	16.5	51.0	24.6	2.7	0.0	0.4	2.0	2.2	0.3	0.0	0.1	0.1	0.1	0.0	100.0	6.2
S22B-SN-H2O2	26.5	49.0	4.7	15.4	0.7	0.5	2.5	0.4	0.0	0.0	0.0	0.0	0.2	0.1	100.0	8.7
S22A-SN-H2O2	33.6	49.4	9.1	5.5	0.6	0.0	1.0	0.3	0.0	0.1	0.0	0.0	0.0	0.0	99.6	9.9
S22A-SI-a-H2O2	23.3	40.0	19.4	14.9	0.9	0.8	0.2	0.0	0.0	0.0	0.1	0.0	0.1	0.3	100.0	9.0
S21-SI-d-burnt	11.4	69.8	16.0	0.7	0.0	0.0	1.4	0.8	0.0	0.0	0.0	0.0	0.0	0.0	100.1	11.2
S21-SI-b-H2O2	27.4	39.6	26.6	1.1	0.1	0.1	3.4	1.4	0.0	0.0	0.1	0.0	0.1	0.0	99.9	9.4
S21-SI-c-H2O2	16.4	58.8	18.3	3.0	0.2	0.0	2.2	0.8	0.1	0.0	0.1	0.0	0.1	0.1	100.1	7.9
S22A-SI-b-H2O2	42.5	43.8	4.0	8.3	0.4	0.2	1.3	0.0	0.0	0.0	0.0	0.0	0.3	0.1	99.9	9.0
S22A-SI-b-raw	23.5	50.1	8.1	11.9	0.9	0.7	2.4	1.6	0.3	0.0	0.0	0.0	0.3	0.1	99.9	6.3
S22A-SI-c-H2O2	37.9	48.0	9.0	3.4	0.5	0.0	0.9	0.0	0.0	0.0	0.0	0.0	0.2	0.0	100.0	9.4

Table S2. Inferred parameters (median and 95 % credible intervals) and metadata for the spectral measurements of the Figure S3. SSL: surface scattering layer. WC: weathering crust.

Spectrum	Algae conc. (10^4 cells mL^{-1})	Cryoconite conc. (mg L^{-1})	Mineral conc. (mg L^{-1})	SSL depth (cm)	SSL SSA (m2 kg^{-1})	WC depth (m)	WC SSA (m2 kg^{-1})	ΔBBA algae	ΔBBA cryoconite	ΔBBA dust	MAE (%)	Date and time	Sky conditions
Upper row, left panel	0.1 [0.0-0.4]	1 [0-4]	55 [22-83]	1.6 [1.5-1.6]	0.20 [0.20, 0.21]	1.9 [1.9-2.0]	< 0.01	0.00 [0.00-0.00]	0.00 [0.00-0.00]	0.00 [0.00-0.00]	1.7	20/07/2021 10:54	Fully cloudy
Upper row, middle panel	8.2 [8.0-8.6]	18 [2-31]	4 [0-17]	2.5 [1.7, 3.0]	0.46 [0.44-0.47]	0.1 [0.0-0.4]	0.02 [0.00-0.25]	0.10 [0.09-0.10]	0.01 [0.00-0.02]	0.00 [0.00-0.00]	1.4	25/07/2022 14:17	Mostly cloudy
Upper row, right panel	1.0 [0.0-3.2]	490 [427-550]	137 [0-287]	1.7 [0.5-3.3]	0.06 [0.06-0.07]	0.4 [0.3-0.7]	0.03 [0.02-0.04]	0.00 [0.00-0.01]	0.13 [0.10-0.17]	0.00 [0.00-0.00]	1.4	22/07/2021 15:31	Fully cloudy
Lower row, left panel	0.0 [0.0-0.1]	1 [0-3]	2 [0-8]	3.1 [2.7-3.5]	0.33 [0.32-0.33]	0.2 [0.2-0.2]	0.12 [0.10-0.15]	0.00 [0.00-0.00]	0.00 [0.00-0.00]	0.00 [0.00-0.00]	1.1	29/07/2021 11:31	Clear with a few clouds
Lower row, middle panel	3.4 [2.9-3.9]	48 [32-60]	372 [338-413]	1.0 [0.9-1.2]	1.69 [1.62-1.74]	0.4 [0.3-0.5]	1.02 [0.74-1.35]	0.08 [0.06-0.09]	0.06 [0.04-0.08]	0.02 [0.01-0.02]	0.7	06/08/2022 15:30	Clear
Lower row, right panel	0.7 [0.0-1.4]	7 [0-17]	178 [113-233]	0.2 [0.0-0.4]	0.06 [0.06-0.07]	0.4 [0.4-0.5]	0.06 [0.06-0.06]	0.01 [0.00-0.02]	0.00 [0.00-0.01]	0.00 [0.00-0.01]	2.7	16/07/2021, 11:33	ND

Table S3. Inferred parameters (median and 95 % credible intervals) and metadata for the spectral measurements of the main Figure 2. SSL: surface scattering layer. WC: weathering crust.

Spectrum	Algae conc. (10^4 cells mL^{-1})	Cryoconite conc. (mg L^{-1})	Mineral conc. (mg L^{-1})	SSL depth (cm)	SSL SSA (m2 kg^{-1})	WC depth (m)	WC SSA (m2 kg^{-1})	ΔBBA algae	ΔBBA cryoconite	ΔBBA dust	MAE (%)	Date and time	Weather conditions
Upper row, left panel	0.4 [0.0-0.8]	1 [0-6]	260 [210-311]	<0.1	/	1.0 [0.9-1.0]	0.01 [0.01-0.02]	0.00 [0.00-0.01]	0.00 [0.00-0.00]	0.00 [0.00-0.01]	11.8	02/08/2022 15:20	Fully cloudy
Upper row, middle panel	0.8 [0.2-1.3]	1 [0-6]	35 [0-74]	3.3 [3.1, 3.4]	0.41 [0.40-0.42]	1.2 [1.1-1.4]	0.01 [0.01-0.02]	0.02 [0.0-0.03]	0.00 [0.00-0.01]	0.00 [0.00-0.00]	1.3	23/07/2021 12:36	Clear
Upper row, right panel	0.5 [0.3-0.6]	1 [0-4]	2 [0-9]	1.6 [1.4-1.7]	1.41 [1.39-1.43]	0.7 [0.6-0.8]	0.85 [0.76-0.95]	0.01 [0.00-0.02]	0.00 [0.00-0.01]	0.00 [0.00-0.00]	0.7	28/07/2022 13:00	Clear
Middle row, left panel	1.7 [0.8-2.7]	52 [30-70]	208 [146-273]	1.3 [1.2-1.4]	0.44 [0.43, 0.46]	0.4 [0.3-0.5]	0.06 [0.05-0.07]	0.03 [0.01-0.04]	0.04 [0.02-0.06]	0.01 [0.00-0.01]	1.5	29/07/2021, 12:24	Mostly cloudy
Middle row, middle panel	1.3 [0.0-3.8]	334 [294-372]	270 [54-425]	4.2 [2.5-5.8]	0.04 [0.04-0.04]	0.8 [0.5-1.2]	0.02 [0.02-0.03]	0.01 [0.00-0.02]	0.11 [0.09-0.13]	0.00 [0.00-0.00]	2.3	17/07/2021 14:45	Clear with a few clouds
Middle row, right panel	4.6 [1.4-7.5]	504 [433-578]	244 [10-467]	2.3 [1.3-3.3]	0.10 [0.10-0.11]	0.7 [0.3-1.0]	0.02 [0.01-0.03]	0.01 [0.00-0.02]	0.11 [0.08-0.14]	0.00 [0.00-0.00]	1.8	22/07/2021 15:35	Fully cloudy
Lower row, left panel	2.6 [2.2-2.9]	9 [0-20]	5 [0-20]	2.6 [2.4-2.9]	0.49 [0.48, 0.51]	0.3 [0.3-0.4]	0.07 [0.06-0.08]	0.05 [0.04-0.05]	0.01 [0.00-0.02]	0.00 [0.00-0.00]	1.0	06/08/2021 13:28	Clear with a few clouds
Lower row, middle panel	7.2 [6.9-7.5]	2 [0-10]	3 [0-14]	2.3 [2.1-2.5]	0.27 [0.26-0.27]	0.2 [0.2-0.3]	0.04 [0.03-0.05]	0.08 [0.08-0.09]	0.00 [0.00-0.01]	0.00 [0.00-0.00]	1.6	06/08/2021 10:41	Clear with a few clouds
Lower row, right panel	15.7 [15.4-16.1]	3 [0-14]	6 [0-29]	1.6 [0.9-2.0]	0.32 [0.31-0.32]	0.04 [0.0-0.6]	0.03 [0.00-0.25]	0.11 [0.11-0.11]	0.00 [0.00-0.00]	0.00 [0.00-0.00]	2.0	06/08/2021, 12:48	Clear with a few clouds

Table S4. Inferred parameters (median and 95 % credible intervals) and metadata for the spectral measurements of the main Figure S4. SSL: surface scattering layer. WC: weathering crust.

Spectrum	Algae conc. (10^4 cells mL^{-1})	Cryoconite conc. (mg L^{-1})	Mineral conc. (mg L^{-1})	SSL depth (cm)	SSL SSA (m2 kg^{-1})	WC depth (m)	WC SSA (m2 kg^{-1})	ΔBBA algae	ΔBBA cryoconite	ΔBBA dust	MAE (%)	Date and time	Weather conditions
Upper row, left panel	2.3 [1.7-2.8]	1 [0-6]	131 [83-181]	<0.1	/	0.7 [0.7-0.8]	0.03 [0.03-0.03]	0.03 [0.02-0.04]	0.00 [0.00-0.00]	0.00 [0.00-0.01]	8.5	16/07/2021, 12:45	ND
Upper row, middle panel	0.7 [0.1, 1.3]	1 [0-6]	119 [76-165]	1.8 [1.7-2.0]	0.23 [0.22-0.23]	0.6 [0.6-0.7]	0.06 [0.05-0.06]	0.01 [0.00-0.02]	0.00 [0.00-0.01]	0.01 [0.00-0.01]	2.0	19/07/2021, 16:50	Fully cloudy
Upper row, right panel	0.6 [0.4-0.8]	2 [0-7]	2 [0-7]	1.8 [1.5-2.1]	0.94 [0.92-0.95]	0.8 [0.7-0.9]	0.80 [0.70-0.94]	0.02 [0.01-0.02]	0.00 [0.00-0.01]	0.00 [0.00-0.00]	0.8	26/07/2022 12:00	Mostly cloudy
Middle row, left panel	2.2 [1.2-3.0]	60 [40-80]	177 [108-245]	1.4 [1.3-1.6]	0.53 [0.51-0.55]	0.5 [0.4-0.6]	0.07 [0.06-0.08]	0.03 [0.02-0.05]	0.05 [0.03-0.07]	0.01 [0.00-0.01]	0.8	02/08/2022 14:54	Fully cloudy
Middle row, middle panel	1.4 [0.1-3.3]	316 [267-358]	113 [0-230]	2.0 [1.4-2.6]	0.09 [0.09-0.10]	0.5 [0.3-0.7]	0.02 [0.02-0.03]	0.01 [0.00-0.02]	0.11 [0.08-0.13]	0.00 [0.00-0.00]	1.9	29/07/2021, 13:04	Fully cloudy
Middle row, right panel	2.2 [0.1-4.3]	420 [358-495]	181 [0-380]	2.6 [1.7-4.0]	0.14 [0.13-0.15]	0.7 [0.3-1.1]	0.05 [0.03-0.08]	0.01 [0.00-0.02]	0.18 [0.14-0.24]	0.00 [0.00-0.00]	1.3	27/07/2021 12:34	Mostly cloudy
Lower row, left panel	1.4 [1.3-1.6]	1 [0-5]	4 [0-17]	2.5 [2.2-2.8]	0.45 [0.44-0.46]	0.2 [0.2-0.2]	0.14 [0.12-0.17]	0.03 [0.03-0.03]	0.00 [0.00-0.01]	0.00 [0.00-0.00]	1.4	02/08/2022 14:00	Fully cloudy
Lower row, middle panel	4.7 [4.2-5.2]	69 [51-88]	9 [0-35]	1.9 [1.3-2.5]	0.43 [0.42-0.45]	0.2 [0.2-0.2]	0.27 [0.18-0.39]	0.07 [0.06-0.08]	0.06 [0.04-0.08]	0.00 [0.00-0.00]	1.3	08/08/2022 11:27	Fully cloudy
Lower row, right panel	11.0 [10.7-11.5]	29 [8-49]	5 [0-21]	2.2 [1.3-3.1]	0.32 [0.31-0.33]	0.1 [0.1-0.2]	0.11 [0.02-0.26]	0.11 [0.11-0.12]	0.01 [0.00-0.02]	0.00 [0.00-0.00]	1.9	08/08/2022 12:31	Fully cloudy

REFERENCES

1. J. Oerlemans, H. Vugts, A meteorological experiment in the melting zone of the Greenland ice sheet. *Bull. Am. Meteorol. Soc.* **74**, 355–366 (1993).

2. R. Shimada, N. Takeuchi, T. Aoki, Inter-annual and geographical variations in the extent of bare ice and dark ice on the Greenland ice sheet derived from MODIS satellite images. *Front. Earth Sci.* **4**, 43 (2016).

3. A. J. Tedstone, J. L. Bamber, J. M. Cook, C. J. Williamson, X. Fettweis, A. J. Hodson, M. Tranter, Dark ice dynamics of the south-west Greenland ice sheet. *Cryosphere* **11**, 2491–2506 (2017).

4. J. Oerlemans, I. Wientjes, An explanation for the dark region in the western melt zone of the Greenland ice sheet. *Cryosphere* **4**, 261–268 (2010).

5. A. Wehrlé, J. E. Box, M. Niwano, A. M. Anesio, R. S. Fausto, Greenland bare-ice albedo from PROMICE automatic weather station measurements and Sentinel-3 satellite observations. *GEUS Bull.* **47**, 10.34194/geusb.v47.5284 (2021).

6. M. Tedesco, S. Doherty, X. Fettweis, P. Alexander, J. Jeyaratnam, J. Stroeve, The darkening of the Greenland ice sheet: Trends, drivers, and projections (1981–2100). *Cryosphere* **10**, 477–496 (2016).

7. S. Feng, J. M. Cook, A. M. Anesio, L. G. Benning, M. Tranter, Long time series (1984–2020) of albedo variations on the Greenland ice sheet from harmonized Landsat and Sentinel 2 imagery. *J. Glaciol.* **69**, 1225–1240 (2023).

8. R.M. Antwerpen, M. Tedesco, X. Fettweis, P. Alexander, W. J. van de Berg, Assessing bare-ice albedo simulated by MAR over the Greenland ice sheet (2000–2021) and implications for meltwater production estimates. *Cryosphere* **16**, 4185–4199 (2022).

9. P.M. Alexander, M. Tedesco, X. Fettweis, R. S. W. Van De Wal, C. J. P. P. Smeets, M. R. Van Den Broeke, Assessing spatio-temporal variability and trends in modelled and measured Greenland ice sheet albedo (2000–2013). *Cryosphere* **8**, 2293–2312 (2014).

10. W. H. Knap, J. Oerlemans, The surface albedo of the Greenland ice sheet: Satellite-derived and in situ measurements in the Søndre Strømfjord area during the 1991 melt season. *J. Glaciol.* **42**, 364–374 (1996).

11. W. Greuell, Meltwater accumulation on the surface of the Greenland ice sheet: Effect on albedo and mass balance. *Geogr. Ann. Ser. B* **82**, 489–498 (2000).

12. A. J. Tedstone, J. M. Cook, C. J. Williamson, S. Hofer, J. McCutcheon, T. Irvine-Fynn, T. Gribbin, M. Tranter, Algal growth and weathering crust state drive variability in western Greenland ice sheet ice albedo. *Cryosphere* **14**, 521–538 (2020).

13. M. L. Yallop, A. M. Anesio, R. G. Perkins, J. M. Cook, J. Telling, D. Fagan, J. MacFarlane, M. Stibal, G. Barker, C. Bellas, A. Hodson, M. Tranter, J. Wadham, N. W. Roberts, Photophysiology and albedo-changing potential of the ice algal community on the surface of the Greenland ice sheet. *ISME J.* **6**, 2302–2313 (2012).

14. M. Stibal, J. E. Box, K. A. Cameron, P. L. Langen, M. L. Yallop, R. H. Mottram, A. L. Khan, N. P. Molotch, N. A. M. Chrismas, F. Calì Quaglia, D. Remias, C. J. P. P. Smeets, M. R. van den Broeke, J. C. Ryan, A. Hubbard, M. Tranter, D. van As, A. P. Ahlstrøm, Algae drive enhanced darkening of bare ice on the Greenland ice sheet. *Geophys. Res. Lett.* **44**, 11463–11471 (2017).

15. J. M. Cook, A. J. Tedstone, C. Williamson, J. McCutcheon, A. J. Hodson, A. Dayal, S. M. Skiles, S. Hofer, R. Bryant, O. McAree, A. McGonigle, J. Ryan, A. M. Anesio, T. D. L. Irvine-Fynn, A. Hubbard, E. Hanna, M. Flanner, S. Mayanna, L. G. Benning, A. van As, M. Yallop, J. B. McQuaid, T. Gribbin, M. Tranter, Glacier algae accelerate melt rates on the south-western Greenland ice sheet. *Cryosphere* **14**, 309–330 (2020).

16. J. C. Ryan, A. Hubbard, M. Stibal, T. D. Irvine-Fynn, J. Cook, L. C. Smith, K. Cameron, J. E. Box, Dark zone of the Greenland ice sheet controlled by distributed biologically-active impurities. *Nat. Commun.* **9**, 1065 (2018).

17. J. McCutcheon, S. Lutz, C. Williamson, J. M. Cook, A. J. Tedstone, A. Vanderstraeten, S. Wilson, A. Stockdale, S. Bonneville, A. M. Anesio, M. L. Yallop, J. B. McQuaid, M. Tranter, L. G. Benning, Mineral phosphorus drives glacier algal blooms on the Greenland ice sheet. *Nat. Commun.* **12**, 570 (2021).

18. I. Wientjes, R. Van de Wal, G.-J. Reichart, A. Sluijs, J. Oerlemans, Dust from the dark region in the western ablation zone of the Greenland ice sheet. *Cryosphere* **5**, 589–601 (2011).

19. M. Musilova, M. Tranter, J. L. Bamber, N. Takeuchi, A.M. Anesio, Experimental evidence that microbial activity lowers the albedo of glaciers. *Geochem. Perspect. Lett.* **2**, 105–116 (2016).

20. Ł. Wejnerowski, E. Poniecka, J. Buda, P. Klimaszyk, A. Piasecka, M. K. Dziuba, G. Mugnai, N. Takeuchi, K. Zawierucha, Empirical testing of cryoconite granulation: Role of cyanobacteria in the formation of key biogenic structure darkening glaciers in polar regions. *J. Phycol.* **59**, 939–949 (2023).

21. N. Takeuchi, S. Kohshima, K. Seko, Structure, formation, and darkening process of albedo-reducing material (cryoconite) on a Himalayan glacier: A granular algal mat growing on the glacier. *Arct. Antarct. Alp. Res.* **33**, 115–122 (2001).

22. S. Wang, M. Tedesco, P. Alexander, M. Xu, X. Fettweis, Quantifying spatiotemporal variability of glacier algal blooms and the impact on surface albedo in southwestern Greenland. *Cryosphere* **14**, 2687–2713 (2020).

23. C. E. Bøggild, R. E. Brandt, K. J. Brown, S. G. Warren, The ablation zone in northeast Greenland: Ice types, albedos and impurities. *J. Glaciol.* **56**, 101–113 (2010).

24. L. Halbach, L.-A. Chevrollier, J. M. Cook, I. T. Stevens, M. Hansen, A. M. Anesio, L. G. Benning, M. Tranter, Dark ice in a warming world: Advances and challenges in the study of Greenland ice sheet's biological darkening. *Ann. Glaciol.* **63**, 95–100 (2022).

25. C. Whicker-Clarke, R. Antwerpen, M. G. Flanner, A. Schneider, M. Tedesco, C. S. Zender, The effect of physically based ice radiative processes on Greenland ice sheet albedo and surface mass balance in E3SM. *J. Geophys. Res. Atmos.* **129**, e2023JD040241 (2024).

26. N. Bohn, T. H. Painter, D. R. Thompson, N. Carmon, J. Susiluoto, M. J. Turmon, M. C. Helmlinger, R. O. Green, J. M. Cook, L. Guanter, Optimal estimation of snow and ice surface parameters from imaging spectroscopy measurements. *Remote Sens. Environ.* **264**, 112613 (2021).

27. L.-A. Chevrollier, A. Wehrlé, J. M. Cook, N. Pirk, L. G. Benning, A. M. Anesio, M. Tranter, Separating the albedo-reducing effect of different light-absorbing particles on snow using deep learning. *Cryosphere* **19**, 1527–1538 (2025).

28. S. M. Skiles, M. Flanner, J. M. Cook, M. Dumont, T. H. Painter, Radiative forcing by light-absorbing particles in snow. *Nat. Clim. Change* **8**, 964–971 (2018).

29. M. G. Flanner, J. B. Arnheim, J. M. Cook, C. Dang, C. He, X. Huang, D. Singh, S. M. Skiles, C. A. Whicker, C. S. Zender, SNICAR-ADv3: A community tool for modeling spectral snow albedo. *Geosci. Model Dev.* **14**, 7673–7704 (2021).

30. H. Langford, A. Hodson, S. Banwart, C. Bøggild, The microstructure and biogeochemistry of Arctic cryoconite granules. *Ann. Glaciol.* **51**, 87–94 (2010).

31. S. M. Skiles, T. Painter, G. S. Okin, A method to retrieve the spectral complex refractive index and single scattering optical properties of dust deposited in mountain snow. *J. Glaciol.* **63**, 133–147 (2017).

32. B. Di Mauro, G. Baccolo, R. Garzonio, C. Giardino, D. Massabò, A. Piazzalunga, M. Rossini, R. Colombo, Impact of impurities and cryoconite on the optical properties of the Morteratsch Glacier (Swiss Alps). *Cryosphere* **11**, 2393–2409 (2017).

33. D. M. Chandler, J. Alcock, J. Wadham, S. Mackie, J. Telling, Seasonal changes of ice surface characteristics and productivity in the ablation zone of the Greenland ice sheet. *Cryosphere* **9**, 487–504 (2015).

34. N. Takeuchi, Optical characteristics of cryoconite (surface dust) on glaciers: The relationship between light absorbency and the property of organic matter contained in the cryoconite. *Ann. Glaciol.* **34**, 409–414 (2002).

35. L.-A. Chevrollier, J. M. Cook, L. Halbach, H. Jakobsen, L. G. Benning, A. M. Anesio, M. Tranter, Light absorption and albedo reduction by pigmented microalgae on snow and ice. *J. Glaciol.* **69**, 333–341 (2023).

36. M. G. Cooper, L. C. Smith, A. K. Rennermalm, C. Miège, L. H. Pitcher, J. C. Ryan, K. Yang, S. W. Cooley, Meltwater storage in low-density near-surface bare ice in the Greenland ice sheet ablation zone. *Cryosphere* **12**, 955–970 (2018).

37. I. T. Stevens, J. M. Cook, L. A. Chevrollier, A. J. Hepburn, A. M. Anesio, L. G. Benning, M. Tranter, The formation and evolution of the supraglacial weathering crust on the Greenland Ice Sheet. EarthArXiv [Preprint] (2025), https://doi.org/10.1017/jog.2026.10146.

38. B. Light, T. C. Grenfell, D. K. Perovich, Transmission and absorption of solar radiation by Arctic sea ice during the melt season. *J. Geophys. Res. Oceans* **113**, C03023 (2008).

39. S. McKenzie Skiles, T. H. Painter, Assessment of radiative forcing by light-absorbing particles in snow from in situ observations with radiative transfer modeling. *J. Hydrometeorol.* **19**, 1397–1409 (2018).

40. M. B. Jensen, T. Turpin-Jelfs, M. Tranter, L. G. Benning, A. M. Anesio, Photophysiological response of glacier ice algae to abiotic stressors. *Front. Geochem.* **2**, 1436488 (2024).

41. L. Halbach, K. Kitzinger, M. Hansen, S. Littmann, L. G. Benning, J. A. Bradley, M. J. Whitehouse, M. Olofsson, R. Mourot, M. Tranter, M. M. M. Kuypers, L. Ellegaard-Jensen, A. M. Anesio, Single-cell imaging reveals efficient nutrient uptake and growth of microalgae darkening the Greenland ice sheet. *Nat. Commun.* **16**, 1521 (2025).

42. Y. Onuma, K. Fujita, N. Takeuchi, M. Niwano, T. Aoki, Modelling the development and decay of cryoconite holes in northwestern Greenland. *Cryosphere* **17**, 3309–3328 (2023).

43. N. Takeuchi, R. Sakaki, J. Uetake, N. Nagatsuka, R. Shimada, M. Niwano, T. Aoki, Temporal variations of cryoconite holes and cryoconite coverage on the ablation ice surface of Qaanaaq Glacier in northwest Greenland. *Ann. Glaciol.* **59**, 21–30 (2018).

44. J. M. Cook, A. Edwards, N. Takeuchi, T. Irvine-Fynn, Cryoconite: The dark biological secret of the cryosphere. *Prog. Phys. Geogr.* **40**, 66–111 (2016)

45. F. Tuzet, M. Dumont, M. Lafaysse, G. Picard, L. Arnaud, D. Voisin, Y. Lejeune, L. Charrois, P. Nabat, S. Morin, A multilayer physically based snowpack model simulating direct and indirect radiative impacts of light-absorbing impurities in snow. *Cryosphere* **11**, 2633–2653 (2017).

46. N. Bohn, E. H. Bair, P. G. Brodrick, N. Carmon, R. O. Green, T. H. Painter, D. R. Thompson, Do we still need reflectance? From radiance to snow properties in mountainous terrain: A case study with the EMIT imaging spectrometer. *Cryosphere* **19**, 1279–1302 (2025).

47. L. Halbach, L.-A. Chevrollier, E. L. Doting, J. M. Cook, M. B. Jensen, L. G. Benning, J. A. Bradley, M. Hansen, L. C. Lund-Hansen, S. Markager, B. K. Sorrell, M. Tranter, C. B. Trivedi, M. Winkel, A. M. Anesio, Pigment signatures of algal communities and their implications for glacier surface darkening. *Sci. Rep.* **12**, 17643 (2022).

48. T. Woods, I. J. Hewitt, A model of the weathering crust and microbial activity on an ice-sheet surface. *Cryosphere* **17**, 1967–1987 (2023).

49. J. M. Cook, A. J. Hodson, A. S. Gardner, M. Flanner, A. J. Tedstone, C. Williamson, T. D. L. Irvine-Fynn, J. Nilsson, R. Bryant, M. Tranter, Quantifying bioalbedo: A new physically based model and discussion of empirical methods for characterising biological influence on ice and snow albedo. *Cryosphere* **11**, 2611–2632 (2017).

50. T. H. Painter, Comment on Singh and others, 'Hyperspectral analysis of snow reflectance to understand the effects of contamination and grain size'. *J. Glaciol.* **57**, 183–185 (2011).

51. D. Stramski, R. A. Reynolds, S. Kaczmarek, J. Uitz, G. Zheng, Correction of pathlength amplification in the filter-pad technique for measurements of particulate absorption coefficient in the visible spectral region. *Appl. Optics* **54**, 6763–6782 (2015).

52. B. H. Toby, R. B. Von Dreele, GSAS-II: the genesis of a modern open-source all purpose crystallography software package. *J. Appl. Cryst.* **46**, 544–549 (2013).

53. A. Wehrlé, AdrienWehrle/earthspy: v0.3.0, Zenodo (2023), https://doi.org/10.5281/zenodo.7498876.

54. A.G. Williamson, A.F. Banwell, I.C. Willis, N.S. Arnold, Dual-satellite (Sentinel-2 and Landsat 8) remote sensing of supraglacial lakes in Greenland, *Cryosphere* **12**, 3045–3065 (2018).

55. C. A. Whicker, M. G. Flanner, C. Dang, C. S. Zender, J. M. Cook, A. S. Gardner, SNICAR-ADv4: A physically based radiative transfer model to represent the spectral albedo of glacier ice. *Cryosphere* **16**, 1197–1220 (2022).

56. B. Briegleb, B. Light, "A Delta-Eddington multiple scattering parameterization for solar radiation in the sea ice component of the Community Climate System Model" (University Corporation for Atmospheric Research, 2007), https://doi.org/10.5065/D6B27S71.

57. A. R. Macfarlane, R. Dadic, M. M. Smith, B. Light, M. Nicolaus, H. Henna-Reetta, M. Webster, F. Linhardt, S. Hämmerle, M. Schneebeli, Evolution of the microstructure and reflectance of the surface scattering layer on melting, level Arctic sea ice. *Elementa Sci. Anthropocene* **11**, 00103 (2023).

58. I. G. Wientjes, R.S.W. Van De Wal, M. Schwikowski, A. Zapf, S. Fahrni, L. Wacker, Carbonaceous particles reveal that late Holocene dust causes the dark region in the western ablation zone of the Greenland ice sheet. *J. Glaciol.* **58**, 787–794 (2012).

59. A. L. Khan, P. Xian, J.P. Schwarz, Black carbon concentrations and modeled smoke deposition fluxes to the bare-ice dark zone of the Greenland Ice Sheet. *Cryosphere* **17**, 2909–2918 (2023).

60. M. G. Cooper, L. C. Smith, A. K. Rennermalm, M. Tedesco, R. Muthyala, S. Z. Leidman, S. E. Moustafa, J. V. Fayne, Spectral attenuation coefficients from measurements of light transmission in bare ice on the Greenland Ice Sheet. *Cryosphere* **15**, 1931–1953 (2021).

61. P. Rowe, M. Fergoda, S. Neshyba, Temperature-dependent optical properties of liquid water from 240 to 298 K. *J. Geophys. Res. Atmos.* **125**, e2020JD032624 (2020).

62. M. D. Hoffman, A. Gelman, The No-U-Turn sampler: Adaptively setting path lengths in Hamiltonian Monte Carlo. *J. Mach. Learn. Res.* **15**, 1593–1623 (2014).

63. J. V. Dillon, I. Langmore, D. Tran, E. Brevdo, S. Vasudevan, D. Moore, B. Patton, A. Alemi, M. Hoffman, R. A. Saurous, TensorFlow distributions. arXiv:1711.10604 [cs.LG] (2017).

64. J. Gorroño, L. Guanter, L. V. Graf, F. Gascon, A framework for the estimation of uncertainties and spectral error correlation in sentinel-2 level-2a data products. *IEEE Trans. Geosci. Remote Sens.* **62**, 1–13 (2024).

65. M. Dumont, O. Brissaud, G. Picard, B. Schmitt, J.-C. Gallet, Y. Arnaud, High-accuracy measurements of snow bidirectional reflectance distribution function at visible and NIR wavelengths—Comparison with modelling results. *Atmos. Chem. Phys.* **10**, 2507–2520 (2010).

66. R. Kumar, C. Carroll, A. Hartikainen, O. Martin, ArviZ a unified library for exploratory analysis of Bayesian models in Python. *J. Open Source Softw.* **4**, 1143 (2019).

67. A. Vehtari, A. Gelman, D. Simpson, B. Carpenter, P. C. Bürkner, Rank-normalization, folding, and localization: An improved $\hat{R}$ for assessing convergence of MCMC (with discussion). *Bayesian Anal.* **16**, 667–718 (2021).

68. S. Tassan, G. M. Ferrari, An alternative approach to absorption measurements of aquatic particles retained on filters. *Limnol. Oceanogr.* **40**, 1358–1368 (1995).

69. D. Stramski, M. Babin, S. B. Wózniak, Variations in the optical properties of terrigenous mineral-rich particulate matter suspended in seawater. *Limnol. Oceanogr.* **52**, 2418–2433 (2007).

70. R. Röttgers, C. Dupouy, B.B. Taylor, A. Bracher, S. B. Wózniak, Mass-specific light absorption coefficients of natural aquatic particles in the near-infrared spectral region. *Limnol. Oceanogr.* **59**, 1449–1460 (2014).
